# Quantitative and qualitative normative dataset for intraepidermal nerve fibers using skin biopsy

**DOI:** 10.1371/journal.pone.0191614

**Published:** 2018-01-25

**Authors:** Nicolas Collongues, Brigitte Samama, Catherine Schmidt-Mutter, Ludivine Chamard-Witkowski, Marc Debouverie, Jean-Baptiste Chanson, Maria-Cristina Antal, Karelle Benardais, Jérôme de Seze, Michel Velten, Nelly Boehm

**Affiliations:** 1 Department of Neurology, University Hospital of Strasbourg, Strasbourg, France; 2 Clinical Investigation Center, INSERM U1434, University Hospital of Strasbourg, Strasbourg, France; 3 Biopathology of Myelin, Neuroprotection and Therapeutic Strategies, INSERM U1119, University Hospital of Strasbourg, Strasbourg, France; 4 Fédération de Médecine Translationnelle de Strasbourg (FMTS), University Hospital of Strasbourg, Strasbourg, France; 5 Faculty of Medicine, Institute of Histology, University of Strasbourg, Strasbourg, France; 6 University Hospital of Besançon, CH Jean Minjoz, Besançon, France; 7 Department of Neurology, University Hospital of Nancy, Nancy, France; 8 Department of Epidemiology and Public Health—EA3430, Faculty of Medicine, University of Strasbourg, Strasbourg, France; University of Würzburg, GERMANY

## Abstract

**Background:**

Skin biopsy is the most relevant tool to diagnose small-fiber neuropathy. A well-documented normal dataset for intraepidermal nerve fiber in the distal leg is required to improve its diagnostic value.

**Methods:**

Three hundred healthy subjects were enrolled in the study, after clinical and biological screening to exclude neurological and systemic pathologies. A distal leg biopsy was taken and intraepidermal nerve fiber density after protein gene product-9.5 immunocytochemistry with brightfield microscopy was determined. Morphological variations of intraepidermal nerve fibers, previously described in small-fiber neuropathies, were analyzed. One hundred biopsies were also analyzed at the ultrastructural level.

**Findings:**

The median number of fibers was lower in men compared to women and decreased with age. Using statistical modeling taking into account age and gender, we calculated the 5th percentile of intraepidermal nerve fiber density as follows: 7.6156–0.0769 x age (years) + 1.5506 x gender (woman = 1; man = 0). We observed a low frequency of large swellings or horizontal branchings but an increasing frequency of small swellings of intraepidermal nerve fibers and irregular distribution along the dermal-epidermal junction with age. Axonal diameter of unmyelinated fibers of the papillary dermis did not vary with age or gender. Ultrastructural analysis also showed that fiber endings in close apposition to Merkel cells should not be mistaken for small-fiber swellings.

**Conclusions:**

Our dataset allows accurate calculation of the normal density of intraepidermal nerve fibers for each year of age and provides original morphological observations that improve the diagnostic value of skin biopsy in the distal leg for small-fiber neuropathy.

## Introduction

Quantification of intraepidermal nerve fiber density (IENFD) at the distal leg using skin biopsy is a widely recognized tool to assess the diagnosis of small-fiber neuropathy (SFN) [1–4]. Contrary to nonconventional electrophysiological tests [5, 6], skin biopsy has proved to be reliable and reproducible: IENFD at the distal leg has high specificity (95%-97%), good sensitivity (45%-80%), a positive predictive value of 92%, and a negative predictive value of 90% [2,7]. Thus, the relevance of a skin biopsy in the diagnosis of SFN has been demonstrated in numerous studies but its wide application encountered some limitations and, in particular, the need for proper normative reference values.

Previous data showed great variations in IENFD as a function of age and gender [8,9], but also according to technical aspects, such as the number of sections analyzed or the immunocytochemical technique used [10,11]. Following two multi-center studies, two normative databases have been made available, one using immunofluorescence and the other using bright-field microscopy, enabling comparisons of IENFD values obtained in hospital expert centers worldwide [1,4]. However, the question of the applicability of these results remains for any center seeking to deliver reliable results and make a significant contribution to the diagnosis of SFN.

This question is prompted by difficulties in the selection of healthy subjects, as this was based on neurological evaluation and a medical questionnaire, without extensive biological screening. Hence, kidney failure, chronic infections or pre-diabetes cannot altogether be ruled out in these subjects, considering that these pathologies may initially be clinically silent. A second limitation of previous studies lies in the ethnicity differences in cutaneous innervation. Indeed, the study by Lauria et al [1] was conducted on European, American, and Asian populations. As a consequence, the great variability in IENFD values measured raises the issue of the heterogeneity of the population studied, and whether such findings are applicable to local populations in expert centers. Finally, the absence of a clinically relevant formulation to assess the IENFD for each year of age and the lack of qualitative data in healthy subjects are two major limitations to using previously reported results in clinical practice.

Considering how important these matters are for patients, we collected skin biopsies from well-documented healthy volunteers and we produced a reliable dataset for normative IENFD with bright-field microscopy, including qualitative aspects. An electron microscopic study was also performed for some biopsies, since we previously showed that ultrastructural modifications are present in the dermis of patients with SFN [12,13].

## Materials and methods

### Standard protocol approvals and patient consent

The study protocol was approved by local (Comité de Protection des Personnes: N°11/612) or national (Agence Nationale du Sécurité du Médicament et des produits de santé: B110819-30) independent ethics committees and performed in accordance with the International Conference on Harmonisation E6 Guideline for Good Clinical Practice, the Declaration of Helsinki principles. All volunteers provided written informed consent before any study-specific procedures were performed.

### Study oversight

The study was conducted at three sites in France. Healthy volunteers were informed of the research protocol by publication of an advert in local newspapers, display of poster in the three referral centers and a webcast. Inclusion criteria were: male or female aged between 20 and 80 years (evenly distributed in ten-year age periods), no history of neurological disorders, normal neurological examination, normal biological assessment (blood and white cell count, coagulation panel, alanine aminotransferase [ALT], aspartate aminotransferase [AST], gamma-glutamyl transferase [GGT], total bilirubin, conjugated bilirubin, blood urea nitrogen, creatinine, clearance of creatinine, sodium, potassium, chloride, fasting plasma glucose, HBV/HBC and HIV serology) in the previous 6 months. Volunteers with a risk factor for peripheral neurological impairment or with a contraindication for skin biopsy were not included.

**Fig 1:**
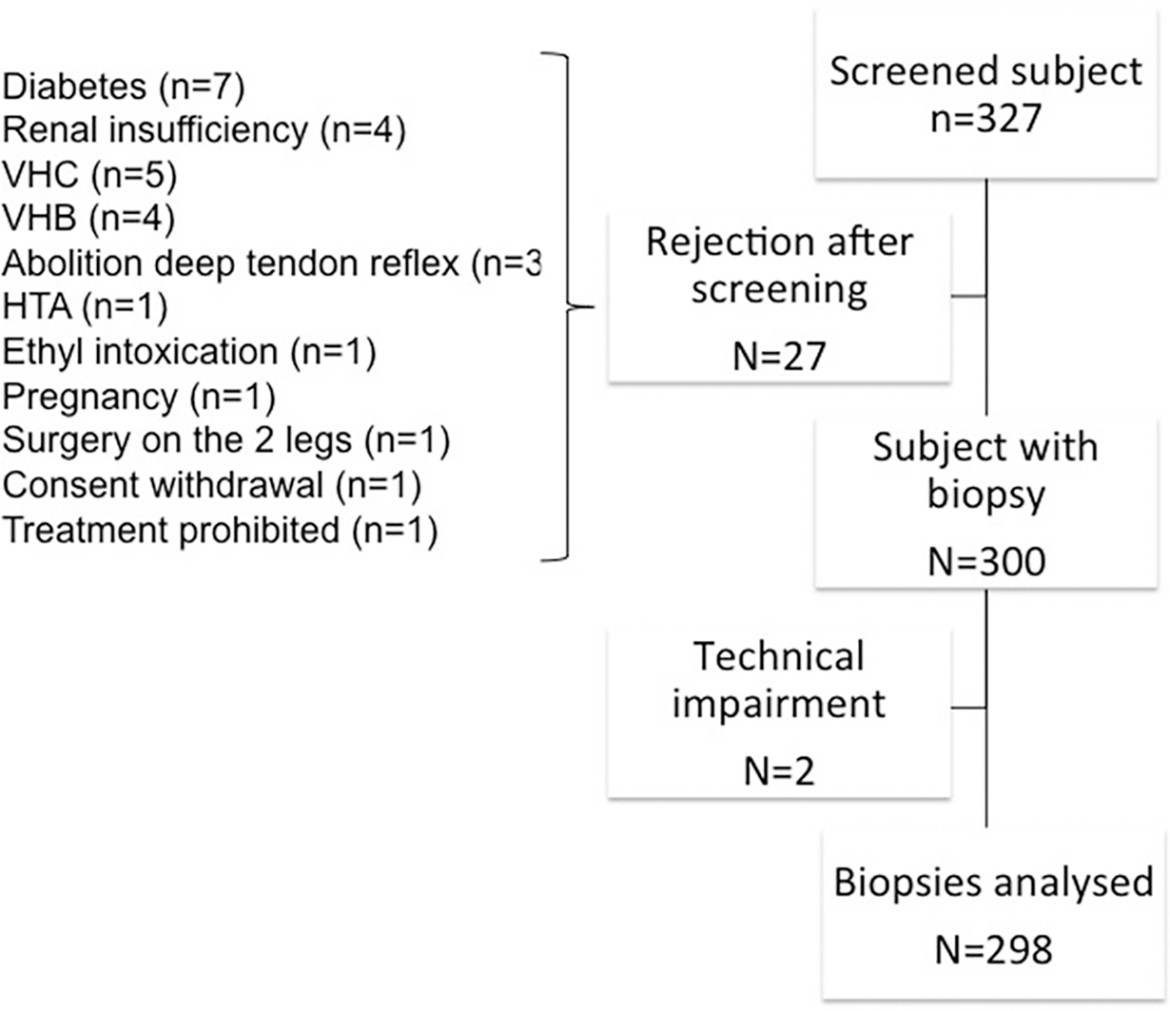
Flow chart indicating the number of healthy subjects and biopsies analyzed in the study. Twenty-seven volunteers failed screening for inclusion in the study. Among them, 23 subjects were at risk of neuropathy for reasons that included diabetes, renal insufficiency, positive serology for HCV or HBV, and absence of deep tendon reflex. Four subjects had a contraindication to skin biopsy (HTA [xylocaine injection], limb surgery, pregnancy, or prohibited therapy).

The selection procedure began by phone, then a neurologist examined volunteers and blood samples were taken. If the volunteer met the inclusion criteria for the study, a skin biopsy was performed 1 week later. Five days after the skin biopsy, volunteers were recalled to collect information on putative side effects. A flow chart summarizes the inclusion process (Fig 1).

### Skin biopsy and intraepidermal fiber counting

#### Skin biopsy

All biopsies were taken from the distal calf, 10 cm above the lateral malleus; the procedure was performed with a disposable 3-mm punch under sterile conditions after local anesthesia with xylocaine; no suture was needed. The biopsies were immediately fixed in freshly prepared 2% paraformaldehyde-lysine-periodate fixative; six to 10 biopsies in each group of age and gender were randomly assigned to both immunocytochemistry and electron microscopy; for these biopsies, a small piece of the specimen was removed from the biopsy 2 hours following the beginning of the fixative period and transferred to glutaraldehyde (2.5% in cacodylate buffer) for further processing (see electron microscopy section); the remaining piece of these biopsies as well as the other entire biopsies were allowed to fix for 24 hours, cryoprotected in 20% sucrose, and frozen. Fifty-microns-thick sections were cut on a cryostat and sections at the beginning and the end of the specimen were discarded, so that only sections with a minimum epidermal length of 2 mm were processed. Sections were alternately assigned to two lots of sections, one of which was immediately used for immunocytochemistry and the other was frozen at -30°C in 20% sucrose for later immunocytochemistry; thus, the sections in each study concerned the whole thickness of the biopsy. A period ranging from two weeks to one year separated the two immunolabeling studies. This procedure was designed to check the reproducibility of the immunocytochemistry technique and the possibility of keeping sections for later use. To generate homogenous results, the same researcher performed all of the immunocytochemistry.

#### Immunocytochemistry

Free-floating sections were incubated in the primary anti-PGP 9.5 antibody (1/10000, Bio-Rad, Marnes-la-Coquette, France) for 24 hours, followed by an overnight incubation in the secondary biotinylated donkey anti-rabbit antibody (1/200, Santa Cruz, Clinisciences, Nanterre, France) and a 4-hour incubation in streptavidin-biotin-peroxidase complex (Vectastain Elite kit, Vector Laboratories, Clinisciences, Nanterre, France); the chromogen was Vector SG (Vector Laboratories, Clinisciences, Nanterre, France). Sections were transferred onto slides and coverslipped with Vectamount AQ (Vector Laboratories, Clinisciences, Nanterre, France).

#### Electron microscopy analysis and measures of dermal axon diameter

The pieces of the biopsies assigned to the electron microscopic study were post-fixed in osmium tetroxide and embedded in Epon. At least three ultrathin sections showing the superficial dermis and the dermal-epidermal junction were analyzed with a Philips EM 208 transmission electron microscope. All the cross sections of the unmyelinated fibers of the papillary dermis were photographed and the diameter of the axons was measured using calibrated ImageJ software (US National Institutes of Health, Bethesda, MD, USA; http://rsb.info.nih.gov/ij/); the mean axon diameter was calculated for each specimen. To validate the measurement of axons on standard ultrathin sections, we also analyzed the dermal and epidermal nerve fibers following PGP 9.5 immunolabeling on one section from two specimens; following immunolabeling, the sections were fixed in glutaraldehyde and osmium tetroxide and embedded in Epon. In one specimen, a cluster of Merkel cells was present.

### Quantitative evaluation of intraepidermal nerve fibers

Only interfollicular epidermis was considered for fiber counting; we also excluded the epidermis above clusters of Merkel cells, present in 10 cases; all fibers crossing the dermal-epidermal junction were counted as previously described [12], yielding a linear density for each section (IENFD = number of intraepidermal nerve fibers (IENFs)/mm). Two histologists blinded to the gender and age of the participants performed counts.

For each biopsy, each operator counted all the sections for both series of immunocytochemistry (4–8 sections for each series). The mean IENFD was calculated separately for each series of immunocytochemistry and for each operator. A comparison of the two series of immunocytochemistry and the two operators was performed. The final result for each case was calculated as follows: first, for every section, the mean of the two values provided by the operators was calculated; second, the median of these results for all the sections belonging to the same series was computed; third, the mean of the two series provided the final result for each case.

### Qualitative evaluation of IENFs

Based on published modifications of IENFs in patients with SFN [14] and on our own experience, we analyzed four modifications of IENFs: presence of lateral and long terminal branchings (excluding branchings perpendicular to epidermal surface), presence of small swellings (1.5–4 μm in diameter), presence of large swellings (more than 4 μm in diameter) and irregular distribution of fibers entering the epidermis along the dermal-epidermal junction. A qualitative score was attributed to each criterion: 0 = absence, 1 = rarely observed, 2 = frequently observed. Two histologists independently determined the scores and sections were reexamined in the event of divergent results.

### Electron microscopy analysis and measures of dermal axon diameter

All the cross sections of the unmyelinated fibers of the papillary dermis were photographed and the diameter of the axons was measured using calibrated ImageJ software (http://rsbweb.nih.gov/ij/); the mean axon diameter was calculated for each specimen. To validate the measurement of axons on standard ultrathin sections, we also analyzed the dermal and epidermal nerve fibers following PGP 9.5 immunolabeling on one section from two specimens.

### Statistical analysis

Unadjusted differences in continuous and categorical variables were assessed for significance using Wilcoxon-Mann-Whitney tests or χ^2^ tests, as appropriate. In order to define reference values for IENFD we used the quantile regression method to analyze the variables influencing the IENFD, concentrating on the median (50th percentile) and the 5th percentile of the distribution [15]. This method offers more flexibility than ordinary regression. Interaction terms were introduced and tested for significance in multivariable models to take into account all variables related to IENFD in univariate analysis. Results were considered statistically significant when p-values were less than 0.05. Therefore, the accuracy of the estimation for the 5th percentile at the mean age of 46 years was ± 3.1 fibers/mm. Analyses were performed with SAS version 9.4 statistical software (SAS Institute Inc., Cary, NC, USA).

All the results are available in the S1 Fig.

## Results

Three hundred twenty-seven subjects were screened for the study. Among them, 27 were rejected on the basis of biological results or clinical examination (Fig 1). Three hundred subjects were included in the study. Demographic and biological data are summarized in Table 1.

**Table 1:** Demographic and biological data of the healthy subjects (n = 300) included in the study.

Ethnicity [C/NC]	Gender [W/M]	Weight (kg) [m±SD]	Age (years) [m±SD]	Height (cm) [m±SD]	BMI [m±SD]	Fasting plasma glucose (g/l) [m±SD]	Uremia (mmol/l) [m±SD]	Creatinine (μmol/l) [m±SD]	CC (ml/mn/UBS) [m±SD]
282/18	147/153	72.4±14.4	45.9±15.2	170±8.9	24.7±4.2	0.9±0.1	5.7 ± 5.1	71.6 ± 15.3	97.5 ± 27.8

C: Caucasian; NC: non Caucasian; W: women; M: men; BMI: body mass index; CC: creatinine clearance; m: mean; UBS: unit of body surface area; SD: standard deviation.

Among the 300 biopsies, 298 were analyzed. Two biopsies were withdrawn from the analysis due to a technical problem during fixation (one biopsy) or immunocytochemistry (one biopsy).

**Fig 2:**
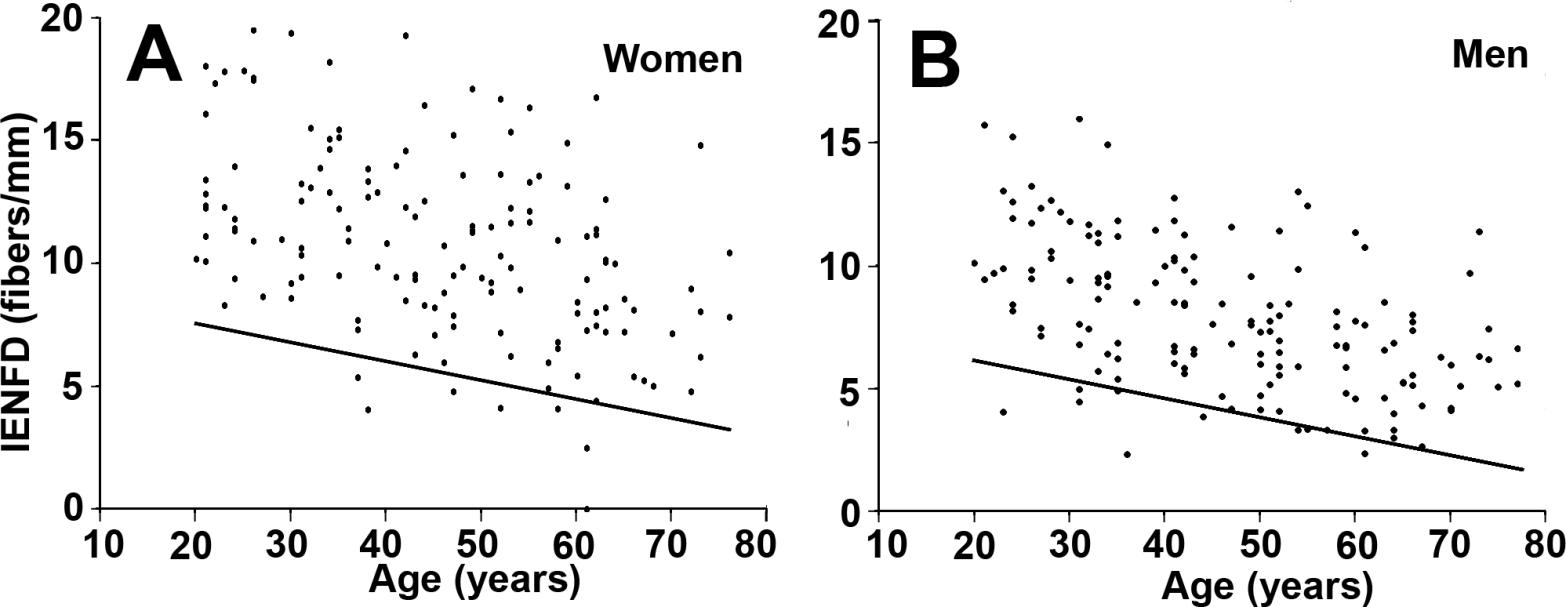
Intraepidermal nerve fiber density. Scatterplot showing intraepidermal nerve fiber density (IENFD) values in the distal leg in healthy subjects according to gender and age (A: women, B: men). Line depicts 5th percentile.

No significant difference in the results was observed between the two operators or between the two immunolabeling studies (p = 0.07); nor did we find any difference when the delay between the two studies was taken into account (p = 0.08). The median IENFD was 9.29 fibers/mm. We observed a lower median number of fibers in men compared to women (7.63 vs 10.74, p <0.0001) and a significant decrease of IENFD with aging (Fig 2A and 2B).

For volunteers of the same age decade, women had an IENFD value that was 1.55 fibers/mm higher when compared to men. Clinical correlations showed no influence of ethnicity but a strong influence of weight, height, and body mass index for the 50th and 5th percentiles of IENFD. Biological correlations showed a decrease in IENFD with increasing uremia, creatinine, and fasting plasma glucose at the 50th but not at the 5th percentile. A lower leverage of the clearance of creatinine was observed with decreasing IENFD, but did not reach statistical significance. In the multivariable analysis, age and gender were correlated with IENFD for the 50th and 5th percentiles, whereas body mass index was correlated only with the IENFD for the 50th percentile. Using a statistical modeling of IENFD adjusted for age and gender, we were able to calculate the 5th percentile of IENFD as follows (Table 2):

5thpercentileofIENFD=7.6156–0.0769xage(years)+1.5506xgender(woman=1;man=0).

**Table 2:** Intraepidermal nerve fiber density (IENFD) normative values for clinical use.

	Women (n = 147)		Men (n = 151)	
Age (years)	Number of subjects	5th percentile IENFD per age span (fibers/mm)	Number of subjects	5th percentile IENFD per age span (fibers/mm)
20–29	25	7.2	24	5.7
30–39	30	6.5	31	4.9
40–49	30	5.7	29	4.2
50–59	28	4.9	31	3.4
60–69	26	4.2	24	2.6
70–79	8	3.4	12	1.8

**Fig 3:**
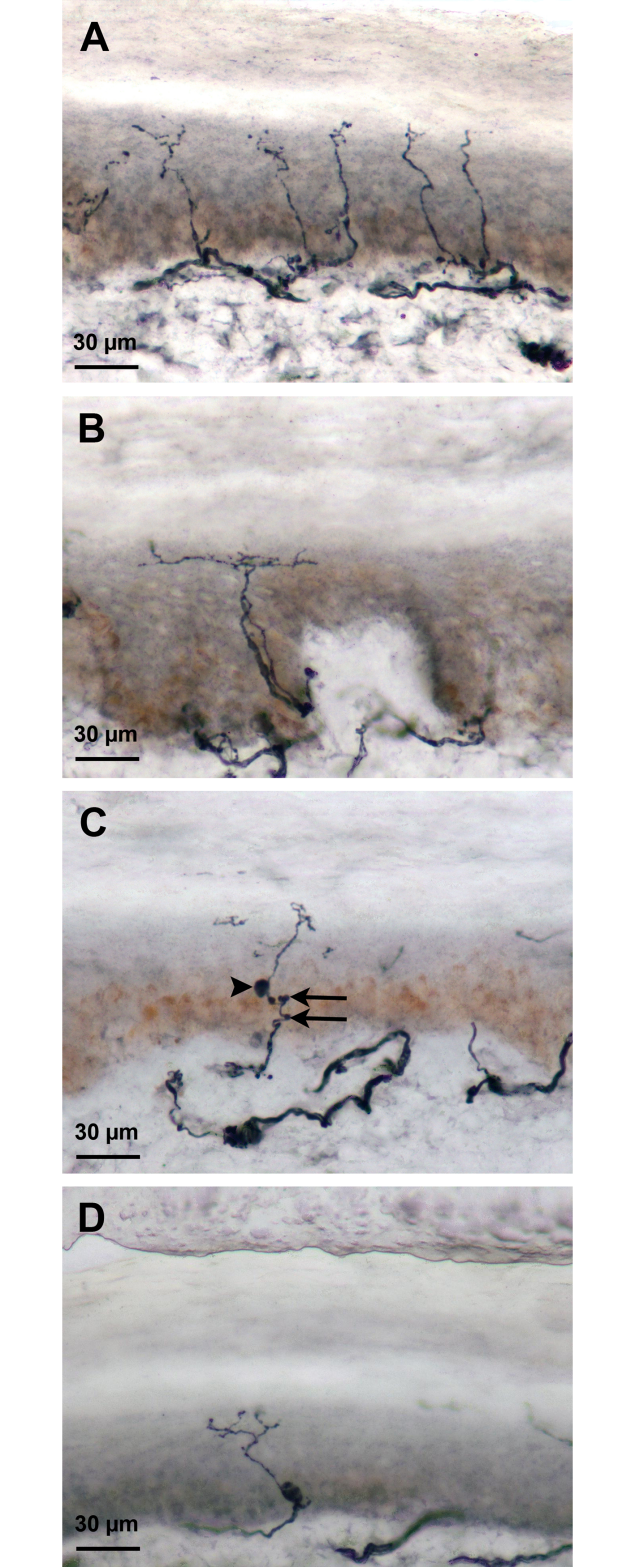
PGP 9.5-immunolabeled intraepidermal nerve fibers (IENFs). A: IENFs regularly distributed in the epidermis in a young man; B: Long horizontal branchings of IENFs in the upper epidermis; C: Small swellings (arrows) and one large swelling (arrow head) on an IENF; D: Isolated large swelling along an IENF.

Qualitative data of IENFs are illustrated in Fig 3A–3D; the score of 2 (abnormality frequently observed) was very rarely attributed.

**Fig 4:**
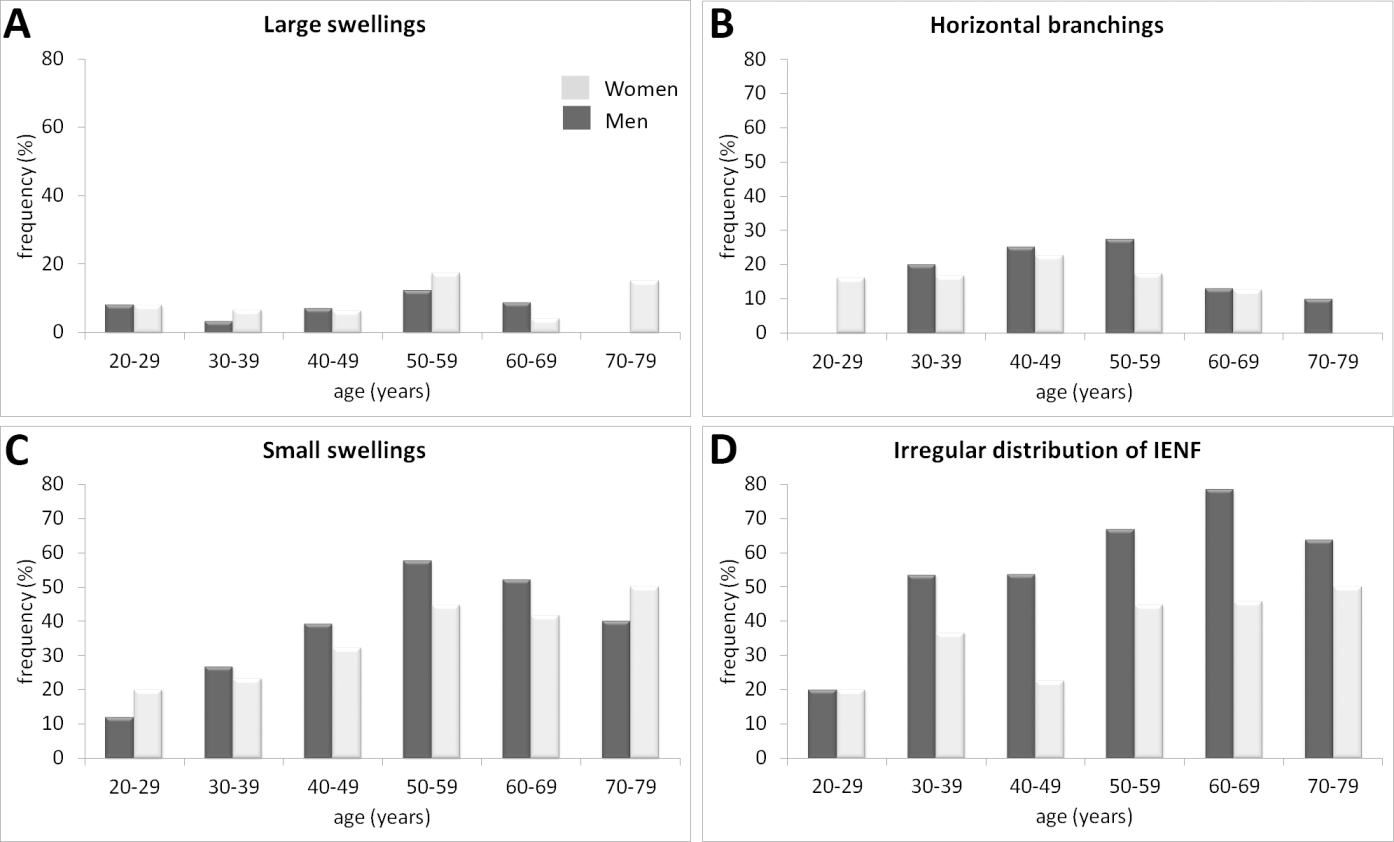
Frequency of intraepidermal nerve fiber (IENF) abnormalities according to age. The frequency represents the percentage of occurrence of morphological abnormalities, including both score 1 and score 2.

In our population of healthy subjects, we did not observe a high frequency of large swellings or branchings of IENFs (Fig 4A and 4B). However, small swellings and irregular distribution of IENFs along the dermal-epidermal junction increased with age (p = 0.01 and p<0.001, respectively) (Fig 4C and 4D).

**Fig 5:**
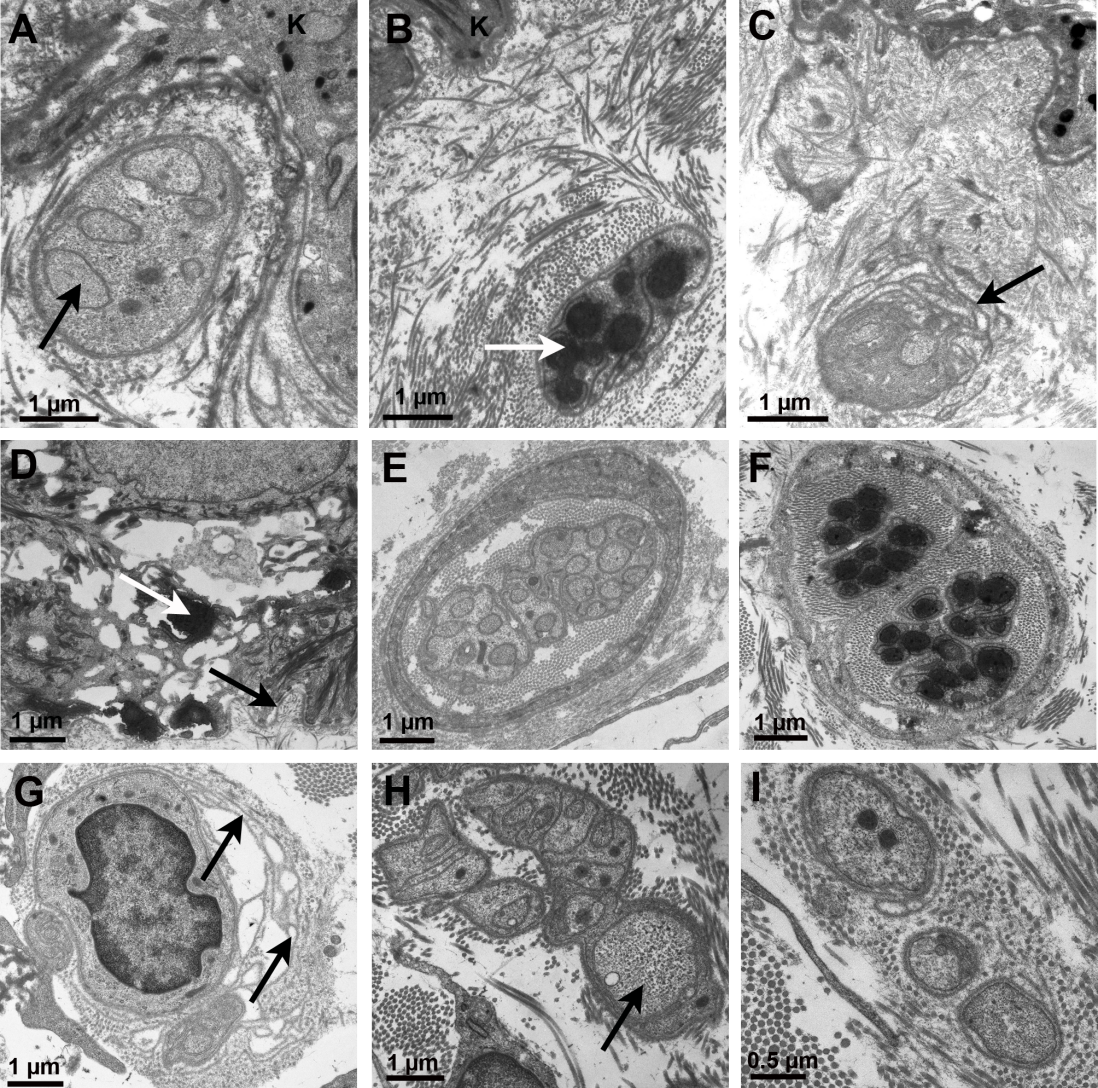
**Ultrastructural observations following standard technique for electron microscopy (A, C, E, G-I) or following PGP 9.5 immunolabeling (B, D, F).** A-B: Several unmyelinated axons (arrows) in a Remak bundle just beneath the dermal-epidermal junction; C: Reduplication of the nerve fiber basal lamina in front of the dermal-epidermal junction (arrow); D: Immunolabeled intraepidermal nerve fibers (IENFs) (white arrow) between basal keratinocytes; the black arrow indicates the basal lamina; E-F: Unmyelinated dermal fibers surrounded by perineural cells; G: Reduplication of nerve fiber basal lamina (arrows); H: The arrow indicates a dilated axon in a superficial dermal unmyelinated nerve fiber; I: Empty Schwann cells. Abbreviation K: basal keratinocyte.

Papillary dermal fibers analyzed at the ultrastructural level were small unmyelinated fibers with one to eight axons surrounded by Schwann cell expansions (Fig 5A and 5B); the basal lamina was often multilayered when close to the epidermal basal lamina (Fig 5C). The diameter of axons ranged from 0.2 to 0.6 μm but the mean diameter (0.35 to 0.42 μm) did not differ with age or gender (p = 0.3). This range of diameter is in accordance with that measured on two immunolabeled sections (mean diameter: 0.38 and 0.41 μm, respectively [Fig 5B]); the cross sections of the IENFs measured in these two cases were larger than those of dermal axons, ranging from 0.5 to 1.4 μm (Fig 5D). The first nerve fascicles surrounded by a perineural layer were observed in the superficial reticular dermis (Fig 5E and 5F). Few modifications of the most superficial fibers were observed: reduplications of the Schwann cell basal lamina at a distance from the epidermal basal lamina (Fig 5G) and very rare swellings of some axons with flocculent material or lysosomes (Fig 5H). A few empty Schwann cells (Fig 5I) were only present in two women of the age 70–79 years group; no collagen pockets or isolated basal laminae were observed.

**Fig 6:**
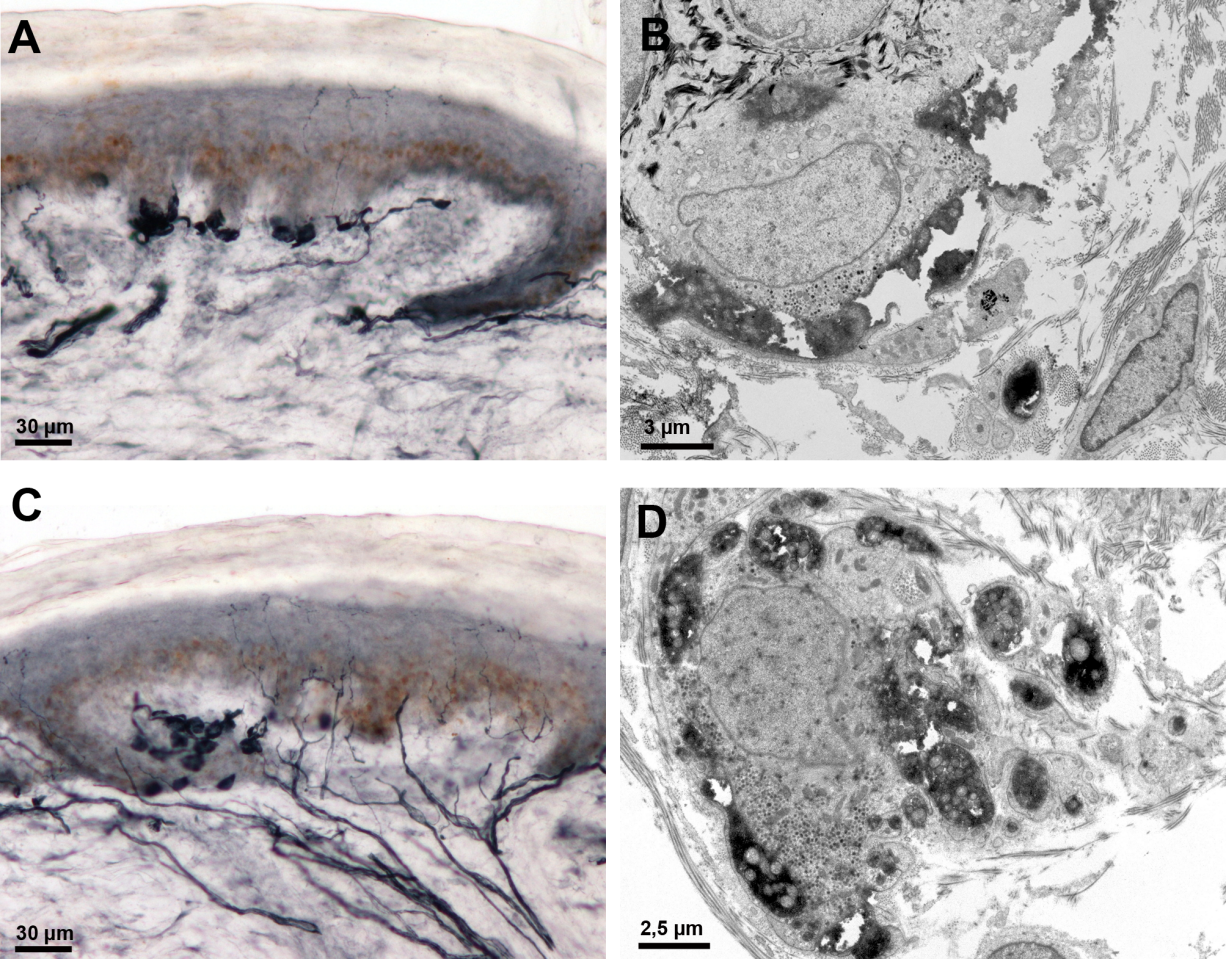
Merkel cells following PGP 9.5 immunolabeling. A-B: Isolated Merkel cells or touch dome in the basal epidermal layer; C-D: Epidermal cluster of Merkel cells; B and D show that only nerve terminals filled with mitochondria are immunolabeled.

Merkel cells were present either as isolated cells or touch dome in the basal epidermal layer (Fig 6A and 6B) or in one case as an epidermal clusters (Fig 6C and 6D). The cells appeared entirely immunolabeled when observed with the optical microscope; however, the ultrastructural analysis showed that only dilated terminal endings filled with mitochondria were labeled; the cytoplasm of Merkel cells was clearly distinguished by its granular content (Fig 6B and 6D) and by desmosomes joining the cell to basal keratinocytes (Fig 6B).

## Discussion

Our study provides a new set of normative data for quantitative and qualitative epidermal innervation in the distal leg. Our cohort of healthy subjects provided a unique opportunity to obtain accurate data for IENFD and this has enabled us to define a pathological threshold for IENFD for each year of age. Analysis of the qualitative results in our population of healthy subjects shows that we did not observe a high frequency of large swellings or branchings of IENFs. As this condition has been described in patients with SFN, our study gives a specific pathological significance to this observation. Our ultrastructural analysis of papillary dermal fibers found that the mean diameter of axons did not differ among groups despite the decrease of IENFD with age and the difference between men and women.

We give an estimation of IENFD in the distal leg, determined using the international recommendation for IENF analysis with bright-field immunocytochemistry, which therefore confers a preliminary guarantee of the applicability of the results [16]. We divided the blinded analysis into two series, which allowed us to test the inter-assay reproducibility of our immunocytochemical method. We concluded that variability was low and we confirmed the reproducibility of the measurements in time. The mean number of sections analyzed by subject was about 10, which is three times more than the number usually performed. It has previously been reported that the variability of estimated IENFD decreases with an increasing number of sections analyzed [10]. Increasing the number of sections is particularly important when analyzing elderly patients’ biopsies as we observed that the regularity of nerve fibers entering the epidermis diminished with age.

Our results in terms of quantitative data are in line with previous publications, but strengthen the validity of IENFD. First, some of these studies were performed before 2005, the year of the first publication of the international recommendation for the use of skin biopsy in SFN [16]. The main paper yielding normal values as a function of age and gender was published in 2010 in a worldwide cohort of healthy subjects[1]. This study was achieved in two stages: the first cohort included 188 volunteers[17] and the second 550 volunteers[1]; the discrepancy in IENFD was particularly noticeable in females, in whom the difference could reach 2.3 fibers/mm for median values ranging from 6.7 to 13.5 fibers/mm. This observation could be partially explained by the heterogeneity of the cohort, which included patients of various origins and without any biological assessment. To avoid these drawbacks, we conducted the analysis of IENFD in a clinically and biologically confirmed cohort of healthy subjects, mainly of Caucasian origin. This approach resulted in the exclusion of 27 subjects. Finally, the method of calculating IENFD in the study by Lauria et al[1] was not detailed and the limitation of IENFD expressed by decade reinforced the difficulty of diagnosing SFN. We therefore propose a model that provides a more accurate estimation of IENFD for each year of age.

In our analysis we excluded the epidermis above the Merkel domes. Merkel cells are usually described as clusters present in the basal layer of hairless skin or in hair shafts. Merkel cells can, however, be present in hairy skin; here, we observed these cells as isolated or clustered in the basal epidermal layer. They could be easily recognized on immunostained sections by their voluminous diameter, which in fact resulted from the staining of nerve ending dilatations surrounding the cells; they should not be mistaken for small-fiber swellings. We excluded these regions because of a general consensus that the nerve terminal connections with these cells result from A-beta terminals, though they may also result from A-delta and C-fibers terminals [18,19].

Morphological alterations of IENFs have been observed in patients suffering from SFNs. Swellings can be used as an indication of future axon degeneration; however, several definitions of swellings are used: axonal enlargements above 1.5 μm [14,20], or at least a two-fold increase in axonal diameter [21–23]. In the present study, based on our experience with patients who often present numerous small and large-sized swellings, we chose to differentiate between these two kinds of axonal enlargement. We observed that multiple small swellings were a common feature with increasing age but that larger swellings were rarely present and not dependent on age, suggesting that only the latter may be of use as an indicator of axonal damage. Abnormal axonal branching has also been used as an indicator of SFN [24, 25], and the absence of excessive branching in our study is in accordance with this. Rajan et al [26], in a model of intracutaneous axotomy, observed that horizontal branching beneath the stratum corneum following axotomy regresses when normal innervation is reestablished, suggesting that they compensate for the absent IEFs. It would be of diagnostic interest to explore whether this mechanism occurs in patients with SFN at the beginning of the disease, when the IENFD is still within the normal range.

We analyzed axonal diameter of the superficial unmyelinated fibers before they enter the epidermis, making sure only sensory fibers were measured. The mean diameter of axons did not differ among groups and there were no morphological alterations of nerve fibers with increasing age, suggesting that any modification that could be observed in pathological situations may be of diagnostic interest; indeed, Doppler et al [27] observed a reduction in mean axon diameter in patients with fibromyalgia syndrome. Swellings of dermal axons were only anecdotal, making this marker of diagnostic interest for SFN [14]. We observed in some cases a redundant basal lamina around Schwann cells with no correlation with age or gender; this may result from environmental cues such as sun irradiation rather than from intrinsic fiber damage [28].

## Conclusions

Skin biopsy is a gold standard for the diagnosis of SFN. Its validity is conditioned by the quality of the normative dataset used to compare IENFs from patients to those of healthy subjects. Our study produced a high quality of quantitative and qualitative information on IENFs in a selected cohort of healthy subjects. We confirm a correlation between IENFD and sex and age and we describe the appearance of Merkel cells after PGP 9.5 immunolabeling, which was distinct from axonal swellings. Lastly, our study revealed that only large swellings and abnormal horizontal axonal branching could be specific of a pathological process in SFN.

## Supporting information

S1 Fig Dataset reporting the characteristics of the healthy subjects in the study. This table includes demographical data (age, ethnic), clinical data (arterial pressure, cardiac frequency, weight, size), date of the clinical visit, biopsy and signature consent but also the biological data and fibers density at the distal leg for each subject.
(XLSX)
